# Is Sacral Neuromodulation a Treatment Option for Primary Bladder Neck Obstruction?

**DOI:** 10.7759/cureus.32931

**Published:** 2022-12-25

**Authors:** Ibrahim Alzahrani, Hossam S El-Tholoth, Ali Alsulihem

**Affiliations:** 1 Urology, Security Forces Hospital, Riyadh, SAU; 2 Urology, Prince Sultan Military Medical City, Riyadh, SAU

**Keywords:** lower urinary tract symptoms, treatment, urodynamic, bladder neck obstraction, sacral neuromodulation

## Abstract

Here, we present the first reported case that used sacral neuromodulation as a treatment option for bladder neck obstruction in a 48-year-old man who presented with a long-standing history of lower urinary tract symptoms such as storage and voiding symptoms unresponsive to conservative and medical treatments, including chemodenervation. The patient was diagnosed with primary bladder neck obstruction (PBNO) using a urodynamic study, voiding cystourethrography, and cystoscopy. Sacral neuromodulation was used because the patient refused bladder neck incision because of the risk of retrograde ejaculation. The patient reported significant improvement in symptoms with no obstructive pattern on follow-up uroflowmetry after six months. The use of alpha-blockers, bladder neck incision, and Botox injection into the bladder neck to treat PBNO has been reported. The successful use of sacral neuromodulation to treat PBNO has not been reported before.

## Introduction

Primary bladder neck obstruction (PBNO) is a functional obstruction caused by abnormal bladder neck opening during voiding. PBNO has several voiding (slow urinary stream, intermittent stream, incomplete emptying) and storage symptoms (frequency, urgency, urgency incontinence, nocturia). Although multiple theories have been proposed, the underlying pathophysiology of PBNO remains unclear. Increased sympathetic nervous activities and excess striated muscle and muscle tone on the bladder neck have been proposed [1,2]. Recently, PBNO was considered bladder neck dysfunction and divided into the following three types: classic high-pressure, low-flow voiding; normal-pressure, low-flow voiding, with narrowing at the bladder neck; and delayed opening of the bladder neck. The treatment options for PBNO in men and women include conservative management, pharmacologic management, and surgical intervention [1-3]. Here, we report the case of a man diagnosed with PBNO who underwent sacral neuromodulation (SNM). To our knowledge, this is the first report on the use of SNM to treat PBNO.

## Case presentation

A 48-year-old man presented with a long-standing history of failure to empty and failure to store lower urinary tract symptoms (LUTS), initially diagnosed with an overactive bladder based on his symptoms and treated at a different facility with 5 mg solifenacin succinate and 50 mg mirabegron, followed by intravesical Botox injection without improvement for one year.

On arrival, the patient’s International Prostate Symptoms Score (IPSS), nocturia, quality of life (QoL), and other parameters were 34/35, 4/5, 6/7, and 5/5, respectively (Table 1). Urine analysis and culture showed no signs of urinary tract infection. Tamsulosin (0.4 mg) was started with partial improvement.

**Table 1:** International Prostate Symptoms Score pre- and post-sacral ‎neuromodulation insertion.‎

Symptoms	Pre	Post
Incomplete emptying	5/5	1/5
Frequency	5/5	5/5
Intermittency	5/5	0
Urgency	5/5	0
Weak stream	5/5	2/5
Straining	5/5	0
Nocturia	4/5	2/5
Total score	34	10
Quality of life	6/6	2/6

**Figure 1:**
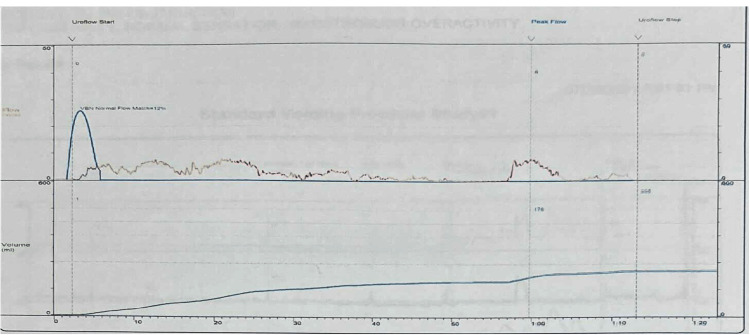
Preoperative uroflowmetry.

Uroflowmetry showed an obstructive pattern, a maximum flow rate of 8 mL/second, a prolonged voiding time, and a prolonged time to maximum flow (Figure 1). A trans-abdominal ultrasound revealed no hydronephrosis, a prostate size of 20 g with a pre-void volume of 529 mL, and a post-void residual volume of 245 mL.

**Figure 2:**
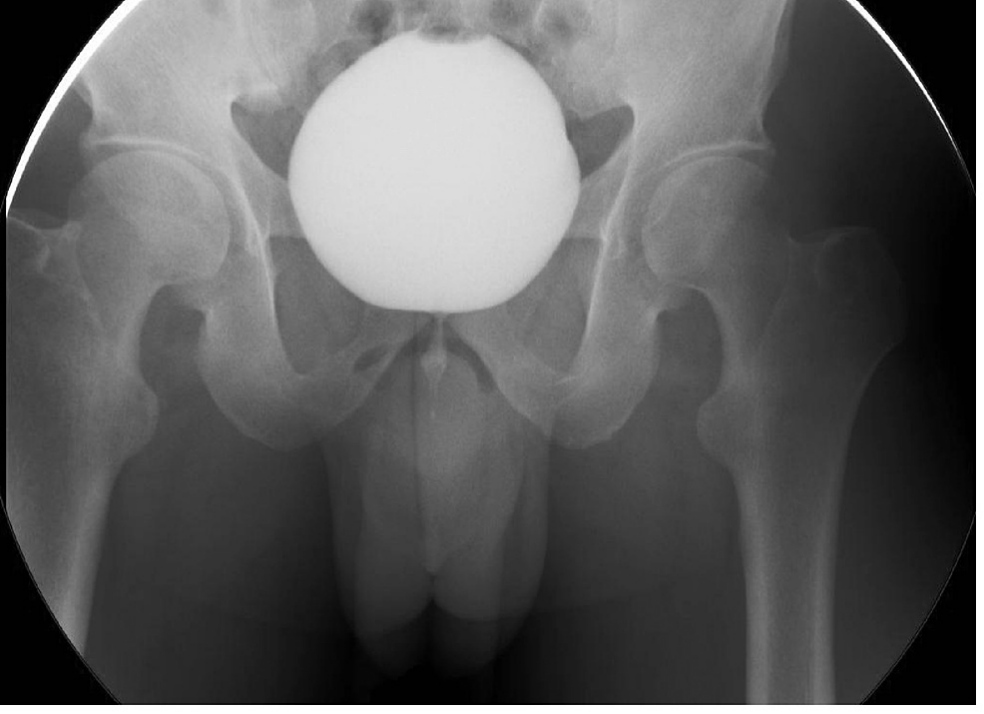
Antegrade voiding cystourethrogram with bladder neck obstruction during voiding.

**Figure 3:**
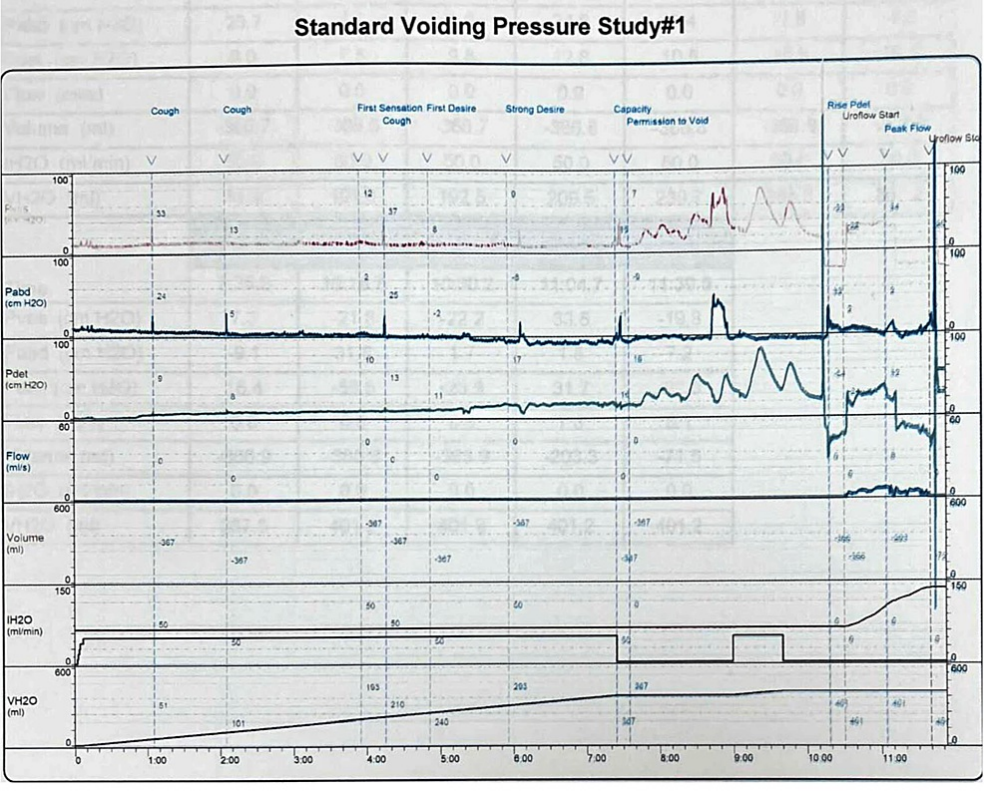
Urodynamic study prior to sacral neuromodulation.

Cystoscopy was negative for urethral stricture, prostatic and bladder tumors, and bladder stones. Narrowing of the bladder neck was identified with minimal trabeculated bladder and no diverticulum giving an impression of bladder neck obstruction. Cystourethrogram showed obstruction of the bladder neck with prolonged bladder emptying (Figure 2). Three months prior to the urodynamic study, the patient stopped anticholinergic and beta-3 agonists. The urodynamic study showed normal capacity, the first sensation was at 163 mL (normal 150-250 mL), a strong desire at 347 mL within the normal range, and compliance with no detrusor overactivity. The patient could not void while the cystometry catheter was in. He generated a detrusor pressure reaching 90 cmH_2_O. The patient developed a very high detrusor pressure without ‎dropping a single drop of urine to be able to calculate the bladder ‎outlet obstruction index. We removed the cystometry catheter, and the patient voided with a low Q max. We were unable to calculate the bladder outlet obstruction index or the bladder contractility index as the cystometry catheter was removed. However, based on the maximum pressure while the catheter was in reached 90 cmH_2_O with 0 mL/second flow, the patient most likely had bladder outlet obstruction (Figure 3). Unfortunately, video urodynamics was not available, but using ‎a combination of urodynamics and voiding cystourethrogram performed ‎by a specialist urologist specialized in functional urology and ‎trained to do video urodynamics, which showed classical images of bladder ‎neck obstruction along with a cystoscopic classical view of bladder ‎neck obstruction, the patient was diagnosed with PBNO.

The tamsulosin dose was increased to 0.8 mg with acceptable partial improvement. It was discontinued because of postural orthostatic tachycardia syndrome (POTS).

**Figure 4:**
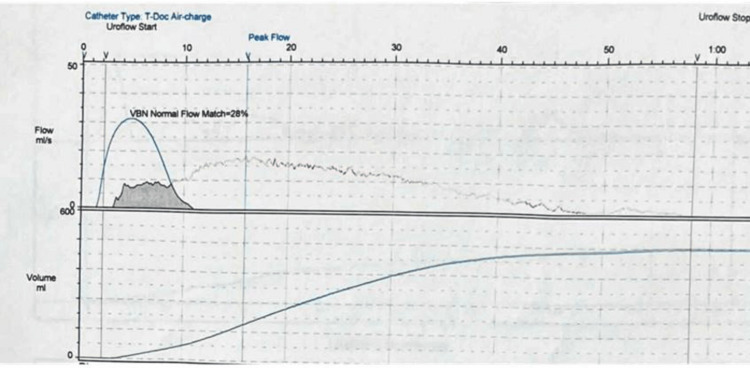
Postoperative uroflowmetry.

The patient was offered both unilateral and bilateral bladder neck incision but he refused any chance of having retrograde ejaculation. The first-stage SNM was then performed. At one month postoperatively, there was a significant improvement in IPSS (34 to 10), QoL (2/6), and preserved antegrade ejaculation (Table 1). Postoperative uroflowmetry showed a normal pattern, a maximum flow rate of 18.8 mL/second, and an improved time to maximum flow (Figure 4, Table 2). Subsequently, we proceed to the second-stage SNM. On the last follow-up after six months, he maintained significant improvement and did not require any medications. Regarding the postoperative urodynamics, we were satisfied with the improvement in the IPSS score and the uroflowmetry. A postoperative urodynamic study was required but the patient refused any more invasive investigations.

**Table 2:** A summary of the uroflowmetry findings pre- and post-sacral neuromodulation.

	Pre	Post
Maximum flow rate (mL/second)	8	18.8
Voiding time (second)	70.8	56.1
Time to maximum flow (second)	57.6	13.8
Voided volume (mL)	204.1	482.1
Post-void residual (mL)	472	0

## Discussion

Although the prevalence of PBNO remains unclear, retrospective studies have shown that the incidence of PBNO in male patients with chronic LUTS ranges from 33% to 55% [3-5].

Regarding medical therapy for PBNO, alpha-blockers remain the main pharmacological treatment. Yang et al. [3] administered 1-2 mg of doxazosin to male patients with PBNO with successful results in 54% of the patients, an IPSS score greater than 50%, and a maximum flow rate of 11.8-15.9 mL/second. Kaplan et al. reported alpha-blocker failure (terazosin and doxazosin) in 31 patients who were misdiagnosed with chronic prostatitis for more than two years and were later diagnosed with PBNO during a video urodynamic study and underwent bladder neck incision [4].

Regarding bladder neck incision, transurethral bladder neck incision remains the surgical treatment of choice for PBNO, with the risk of antegrade ejaculation postoperatively. In the study by Trockman et al. [5], 18 out of 36 patients with PBNO on alpha-blockers did not improve and underwent bilateral transurethral bladder neck incision. Antegrade ejaculation was preserved in 73% of the cases, and the mean maximum flow rate increased from 8.2 mL/second to 26.7 mL/second. Kaplan et al. [4] performed unilateral bladder neck incisions in 31 patients. In 30 men, the mean maximum flow rate increased from 9.2 mL/second to 15.7 mL/second. There were no reports of retrograde ejaculation [4,5].

Hennus et al. [6] followed up 40 patients (median age at first intervention was 4.7 and 19.6 years) who underwent unilateral transurethral bladder neck incision. Antegrade ejaculation was preserved in all patients. However, 10.8% of the patients had reduced ejaculatory volume [6].

Botulinum toxin injection at the level of the prostate, urethra, and bladder neck is a novel treatment for LUTS caused by benign prostatic hyperplasia and dysfunctional voiding [7,8]. Sacco et al. [9] reported on 35 patients with PBNO refractory to alpha-blockers who underwent Botox injection into the bladder neck. Botox (200 U) was diluted in 4 mL of saline (50 U/mL) and injected at four sites (3, 6, 9, and 12 o’clock, 1 mL/site) into the bladder neck at approximately 1 cm from the bladder-neck rim. Postoperative follow-up of 29 patients at two, six, nine, and 12 months showed improvement of the IPSS (from 21.9 at baseline) to 7.8, 10.3, 16.6, and 19, respectively, and maximum flow rate (from 7.8 mL/second at baseline) to 16.9, 15.5, 12.7, and 8.6 mL/second, respectively. Adequate patient satisfaction with preserved antegrade ejaculation was achieved with the treatment, although it was not statistically significant at 12 months postoperatively. Therefore, repeated treatment is required for long-term effectiveness.

Ip et al. [10] reported the outcomes of using Botox to treat PBNO in 13 patients with PBNO with a median maximum flow rate of 14 mL/second. Botox (100 U), diluted in 4 mL of normal saline (25 U/mL) was injected into the bladder neck at four sites (3, 6, 9, and 12 o’clock). Of the 12 patients followed up, nine (75%) reported subjective improvement and three (25%) reported no improvement. Seven patients received a second dose of Botox injection. The median interval time after the first injection was 7.4 months. The third dose of Botox injection was administered to four patients. The median interval time after the second injection was 9.2 months. The fourth and fifth doses were received by two patients after 12 and 17.7 months and 10.3 and 9.7 months, respectively. The IPSS score and maximum flow rate were not reported at baseline or in the serial follow-up postoperatively [10].

Serpilli et al. [11] proposed a new surgical approach to treat PBNO, which consisted of drilling the bladder neck using a laser to make several holes without muscle fiber disruption. Five patients showed improvement in LUTS and preservation of antegrade ejaculation. After 12 months of follow-up, the mean IPSS score and maximum flow rate improved from 17.2 to 3.2 and 8.78 to 18.7 mL/second, respectively. Table 3 summarizes the subjective and objective measures of each reported treatment modality.

**Table 3:** A summary of the differences in pre- and post-treatment symptoms and maximum flow rate ‎‎(Q max) using several reported treatment options. BNI = bladder neck incision; BN = bladder neck

Series	Approach	Mean symptoms score		Mean maximum flow rate (mL/second)	
		Pre	Post	Pre	Post
Yang et al. [3]	Doxazosin	18.3	11.6	11.8	15.9
Kaplan et al. [4]	Unilateral BNI	16.4	6.4	9.2	15.7
Trockman et al. [5]	Bilateral BNI	17.1	4.3	8.2	26.7
Serpilli et al. [11]	BN drilling	17.2	3.2	8.78	18.7
Sacco et al. [9]	BN Botox injection	21.9	10.3	7.8	15.5

Traditionally, medical therapy and bladder neck incision are the initial and definitive treatment options, respectively. Young patients, who are concerned about ejaculatory function and fertility, may be reluctant to undergo a procedure with the risk of ejaculatory dysfunction. Botulinum toxin injection (an alternative option) results in acceptable and temporary relief of symptoms for several months. However, repeated injections are needed to maintain the results [7-10]. Botulinum toxin injection into the bladder neck might affect ejaculatory function, but this has not been reported. To our knowledge, this is the first case reported on the use of SNM to treat PBNO.

The Food and Drug Administration (FDA) has approved SNM for treating overactive bladder, non-obstructive urinary retention, and bowel dysfunction, and other non-FDA approved indications include chronic pelvic pain and bladder pain syndrome [12-14] SNM has a positive impact on ejaculatory function compared to alpha-blockers and bladder neck incision [15,16].

SNM has been traditionally excluded as a treatment option for PBNO because it might be considered an anatomical obstruction (contraction and elevation of the bladder neck) [17,18]. SNM is usually avoided if abnormalities are found during cystoscopy, although PBNO is defined as a functional cause of bladder outlet obstruction (BOO) [19]. We suggested this modality depending on the mechanism of action of SNM which is not clearly well-known. It has been used in Fowler syndrome, which is a non-relaxation of the external sphincter. Bladder neck obstruction is a functional obstruction caused by the contraction of bladder neck fibers that often responds to alpha-blockers. Because the patient had frequency and urgency and had tried medical therapy and Botox injection without benefit and rejected bladder neck obstruction, we viewed it as functional obstruction just like Fowler syndrome and considered SNM. We informed the patient that this is an unorthodox treatment option and has never been tried before, following which the patient agreed to proceed with the first stage. SNM has never been studied as a treatment option for PBNO. Botulinum toxin injection and drilling of the bladder neck have been reported as alternative treatment options because bladder neck incisions can cause sexual dysfunction.

We used SNM because the patient refused bladder neck incision to avoid the risk of retrograde ejaculation. SNM was offered because it was considered a functional obstruction because the patient responded partially to medical therapy. The patient reported significant improvement of the symptoms with no obstructive pattern on follow-up uroflowmetry and preserved antegrade ejaculation. At six months, the patient reported high satisfaction.

This report has several limitations. It is a case report of a single patient and further studies are warranted to evaluate the long-term results on several patients to determine the overall success rate of SNM for treating PBNO. Electromyography and video urodynamics, the gold standard methods to diagnose PBNO and exclude dysfunctional voiding and pelvic floor dysfunction as the causes of BOO, were not done. Cystoscopy, voiding cystourethrogram (VCUG) performed by a specialized urologist in voiding dysfunction, and conventional urodynamics have been used to diagnose BOO and localize the obstruction at the bladder neck.

Additionally, video urodynamics can sometimes be non-diagnostic, as many patients cannot void during the test due to situational inability [20]. Cystoscopy can show some findings that might suggest bladder neck obstruction (a contracted and elevated bladder neck with a small prostate) [17]. The findings of cystoscopy, VCUG, and urodynamic studies, with the partial symptomatic improvement with alpha-blockers, support our final diagnosis of PBNO. We believe that SNM might be an alternative treatment for bladder neck obstruction in men (and potentially women) with minimal sexual side effects. Further studies with more patients and long-term follow-ups are needed to confirm our findings and to evaluate its efficacy in women.

## Conclusions

SNM might be considered for treating PBNO to achieve symptom resolution after the failure of medical therapy without the risk of ejaculatory dysfunction, as seen in this case. Further studies are needed to assess the treatment outcomes and long-term efficacy of this treatment.
